# CLINICIAN TYPE AND SETTING OF CARE DELIVERED TO MEDICARE BENEFICIARIES WITH DEMENTIA IN THE U.S

**DOI:** 10.1093/geroni/igae098.4286

**Published:** 2024-12-31

**Authors:** Donovan Maust, Rachel Davis, Ulrike Muench, Steven Marcus, Joanne Spetz

**Affiliations:** University of Michigan, Michigan, United States; University of Michigan, Ann Arbor, Michigan, United States; UCSF, San Francisco, California, United States; University of Pennsylvania, Philadelphia, Pennsylvania, United States; University of California, San Francisco, San Francisco, California, United States

## Abstract

Improving care coordination for persons living with dementia (PLWD) requires understanding of the types of clinicians delivering care and the settings in which they practice. We identified all beneficiaries with dementia in traditional Medicare in 2019. We used the Medicare Carrier file—i.e., where bills for care provided by individual clinicians are recorded—to identify clinicians providing care to PLWD, which we then linked to the National Plan & Provider Enumeration System (NPPES). Using the Carrier and NPPES files, we characterized the workforce providing care to PLWD, including specialty and licensure (e.g., primary care physician, neurologist, nurse practitioner), setting of care delivery, and whether the clinician included a dementia diagnosis in the billing information. Overall, 1,934,318 PLWD received care from 783,225 unique clinicians. The most common settings of care were office (74.8% of PLWD), emergency room (63.9%), inpatient hospital (52.1%), and skilled nursing facility (37.1%). 87.0% of PLWD received care from a primary care physician, 62.9% from a nurse practitioner, and 33.1% from a physician assistant. Among all the clinicians that care for PLWD, 20.3% are primary care physicians, 15.3% nurse practitioners, and 9.0% physician assistants. 2.4% are psychiatrists, 1.7% neurologist, and just 0.5% are geriatric subspecialist physicians. For clinicians providing care to PLWD, the majority provide care in the outpatient setting, followed by hospital-based care. Programs to improve care coordination for PLWD, such as GUIDE Model programs, must account for delivery across multiple health care settings, recognizing that geriatric subspecialists comprise an extremely small proportion of the PLWD-serving workforce.

